# The Curvilinear Relationship between State Neuroticism and Momentary Task Performance

**DOI:** 10.1371/journal.pone.0106989

**Published:** 2014-09-19

**Authors:** Jonas Debusscher, Joeri Hofmans, Filip De Fruyt

**Affiliations:** 1 Faculty of Psychology and Educational Sciences, Vrije Universiteit Brussel, Brussels, Belgium; 2 Department of Developmental, Personality and Social Psychology, University of Ghent, Ghent, Belgium; Institutes for Behavior Resources and Johns Hopkins University School of Medicine, United States of America

## Abstract

A daily diary and two experience sampling studies were carried out to investigate curvilinearity of the within-person relationship between state neuroticism and task performance, as well as the moderating effects of within-person variation in momentary job demands (i.e., work pressure and task complexity). In one, results showed that under high work pressure, the state neuroticism–task performance relationship was best described by an exponentially decreasing curve, whereas an inverted U-shaped curve was found for tasks low in work pressure, while in another study, a similar trend was visible for task complexity. In the final study, the state neuroticism–momentary task performance relationship was a linear one, and this relationship was moderated by momentary task complexity. Together, results from all three studies showed that it is important to take into account the moderating effects of momentary job demands because within-person variation in job demands affects the way in which state neuroticism relates to momentary levels of task performance. Specifically, we found that experiencing low levels of state neuroticism may be most beneficial in high demanding tasks, whereas more moderate levels of state neuroticism are optimal under low momentary job demands.

## Introduction

Because of its theoretical and practical significance, the relationship between personality and performance has been studied extensively [1]. Whereas the results of these studies have undisputedly demonstrated that personality relates to performance at work [2], almost all of them –including some meta-analytic ones– also concluded that the relationship is relatively weak [3] – [5]. Drawing on these results, some authors have urged practitioners not to rely too heavily on personality measures, while others have simply discouraged their use in personnel selection [6].

Despite these early pessimistic findings, recent studies have shown that a number of methodological issues may account for these weak relationships. One of the most important findings at this point was the realization that modeling the personality-performance relationship in a linear way can seriously misrepresent the true relationship [2], [7] – [10]. Indeed, a number of recent studies have demonstrated that trait conscientiousness and trait neuroticism relate to overall task performance in a curvilinear way [2], [8], [10].

However, a limitation of these studies is that they have exclusively focused on trait differences, whereas organizational scholars have progressively moved towards an integrative approach to personality that recognizes the importance of both personality traits (i.e., between-person differences in personality) and states (i.e., within-person differences in personality) [11]. Such an integrative approach is important in that it can explain the seeming contradiction that people do exhibit stability of cognition, affect, and behavior over time, while at the same time they also vary across occasions. Moreover, as Judge and colleagues [11] argue, personality-related states –or short-term fluctuations in personality– and personality traits are two sides of the same coin because the latter focus on the stable, whereas personality-related states focus on the variable component of personality. That both components of personality are important for performance is illustrated by the fact that we not only expect a surgeon to be calm and in control on average (i.e., low trait neuroticism), but also that (s)he remains calm in both emergency and routine surgeries (i.e., low state neuroticism).

The aim of the present study is to add to the integrative approach to personality by examining whether the curvilinear personality-performance relationship found at the trait level also holds at the state level. In other words, we will explore whether the relationship between stable, between-person differences in personality and stable, between-person differences in performance generalizes to the momentary, within-person level. Note that this issue is far from trivial as relationships at the static, between-person level do not readily apply to the dynamical, within-person level [12]. As such, it remains an open question how increases/decreases in one's state personality relate to increases/decreases in one's task performance.

To address this question, we focused on the within-person relationship between momentary task performance and state neuroticism, or the momentary tendency to experience negative emotions [13]. The reason for choosing neuroticism is that –together with conscientiousness– it is considered one of the best personality predictors of performance [1], and that the amount of within-person variability in neuroticism is often as large as or larger than the between-person variability [14] – [16]. Moreover, because previous research has suggested that the curvilinear relationship between trait personality and performance varies as a function of characteristics of the job [2], [8] we also examined the moderating effect of within-person fluctuations in work pressure and task complexity on the curvilinear within-person relationship between neuroticism and task performance. Both work pressure and task complexity are typical job demands, which have an effect on a vast amount of work-related outcome variables [17] – [19]. In line with Mangos and Steele-Johnson [19] we define task complexity as an individual's perception of how complex a task is. Work pressure in turn is defined as the amount of work a person has, combined with the required speed to fulfill it [20].

By focusing on the within-person relationship between momentary task performance and state neuroticism, our study seeks to contribute to the knowledge and understanding of the neuroticism-performance link in at least two ways. At the theoretical level, we aim to shed light on the processes through which people's behaviors on the job are affected by changes in their level of state neuroticism. Whereas previous (cross-sectional, between-person) studies have addressed this question for stable, between-person differences in neuroticism and task performance [2], this question has not yet been addressed for their dynamical, within-person counterparts. Moreover, by examining the moderating effect of within-person fluctuations in job demands, we try to come to a better understanding of the work conditions that potentially qualify the curvilinear state neuroticism-task performance relationship. On a practical level, our study aims to provide guidance for the everyday management of employees and the conditions they are working in, as it has already been shown that fluctuations in state neuroticism partly result from events that happen at work [13], [21].

### The curvilinear relationship between neuroticism and task performance

In a recent multi-study paper, Le and colleagues [2] found support for a curvilinear relationship between trait neuroticism and overall task performance. In particular, and in line with the Yerkes-Dodson law [22], they found that moderate levels of trait neuroticism were associated with higher levels of task performance than low and high levels of trait neuroticism. The rationale behind this curvilinear relationship was that people high on trait neuroticism were prone to experiencing negative emotions [23], which, according to the ‘attention allocation mechanism’, helped them narrowing down the range of cue utilization [24], and this in turn helped them to focus on what they were doing. Thus, people scoring high on trait neuroticism show high negative emotionality, and this heightened level of negative emotionality helps them to exclude irrelevant task cues and focus on the relevant ones, which in the end promotes performance. At the same time, high levels of trait neuroticism are not always beneficial because beyond a certain point the attention focus becomes so narrow that not only the irrelevant, but also relevant cues are discarded [2]. As a result, performance is hypothesized to be low at low and high levels of emotionality, while it increases when the level of neuroticism is moderate. Note that this prediction is in line with the meta-theoretical too-much-of-a-good-thing principle [25] which states that high levels of antecedent variables that are widely accepted to lead to desirable outcomes (such as emotional stability) are often counterproductive.

### The curvilinear neuroticism–task performance link: from between to within individuals

Whereas the studies of Le and colleagues [2] provided insights into the nature of the neuroticism-performance relationship at the between-person level, it remains an open question whether this relationship also holds at the within-person level. As argued above, the reasoning at the between-person level is that stable individual differences in neuroticism are reflected in stable individual differences in attention focus, which in turn are reflected in stable individual differences in general task performance. Whereas it is indeed true that people differ from each other in their default level of neuroticism, a large body of research in the personality domain has shown that people also fluctuate considerably from situation to situation [14] – [16]. Moreover, the same reasoning holds for task performance, that is, task performance is not stable across time and tasks, but instead fluctuates in an episodic manner [26]. An important subsequent question is then whether these intra-individual fluctuations in state neuroticism are linked to intra-individual differences in task performance in the same –curvilinear– way. This question is an important one as it sheds light on the processes through which people's behaviors on the job are affected by changes in their level of state neuroticism. Moreover, it is a well-known fact that mechanisms that hold at the between-person level do not readily apply to the within-person level [12]. For example, research in the domain of exercising and health has shown that the risk of having a heart attack is lower for people who exercise more. Yet, at the same time, an individual is more likely to experience a heart attack while exercising (e.g. [27] – [28]). Similarly, Vancouver and colleagues [29] have shown that the relationship between performance and self-efficacy reverses when going from the between- to the within-person level. Because of this reason, between-person, cross-sectional studies are not well suited to study the within-person neuroticism-momentary task performance link [30]. Rather, to examine such within-person relationships, data collection methods such as diary studies and experience sampling studies are needed. In diary studies [31], employees report at the end of their working day their previous-day level of neuroticism and task performance, while in experience sampling studies [30] they are queried, multiple times a day, about their momentary levels of neuroticism and task performance. Because in both diary and experience sampling studies the same subject is measured at different points in time and in different situations, both data collection methods allow to study within-person processes. A major advantage of these data collection methods is that one can obtain information on peoples' affective states, cognitions, and behaviors throughout the course of their actual, daily lives, thereby capturing life “as it is lived” [31]. By using both dynamic data collection methods (i.e., diary and experience sampling research), the present study will contribute to the understanding of the neuroticism-task performance relationship at the within-person level.

We hypothesize that within-person fluctuations in state neuroticism are linked to within-person fluctuations in task performance in a curvilinear way. In particular, increases in state neuroticism are linked to the narrowing of attention, which helps people to focus on what they are doing, and subsequently promotes task performance. However, whereas increases in state neuroticism might be beneficial at the lower levels of neuroticism, they become counterproductive at the higher levels of neuroticism because at these levels, relevant cues are disregarded as well, which decreases task performance. Therefore, we hypothesize that:

#### Hypothesis 1

Within-person variation in state neuroticism is linked to within-person variation in task performance through a curvilinear (inverted U-shaped) function.

### The curvilinear neuroticism–task performance link: the moderating role of within-person variation in job demands

Previous work has suggested [8] and demonstrated [2] that the curvilinear relationship between personality and task performance may depend on the characteristics of the job. For example, Le and colleagues [2] showed that task complexity moderated the curvilinear relationship between trait neuroticism and performance such that the relationship was more curvilinear for low than for high complex jobs. However, these studies have traditionally conceptualized job characteristics as relatively constant for any one person. However, it is a well-known fact that, in their daily working lives, people are confronted with a continuously changing set of events [32]. We contribute beyond the usual treatment of job characteristics by examining the moderating effect of within-person fluctuations in job demands on the curvilinear within-person state neuroticism-task performance relationship. By doing so, we tested whether the moderating effect found at the between-person level also holds at the within-person level. Note that studying boundary conditions is crucial for a thorough understanding of the within-person neuroticism-task performance relationship as moderators may affect the direction and even the form of the relationship [33].

In the present paper, we focused on two of the most commonly used and studied job demands, namely work pressure and task complexity [17] – [18]. The reason for choosing these two demands is twofold. First, they have been studied extensively, also in relationship to the personality-performance relationship (e.g. [2]). Second, work pressure and task complexity can be conceptualized as both job challenges and job hindrances, which implies that they capture the full range of job demands [34].

Regarding the moderating role of work pressure and task complexity, we believe that tasks carried out under high job demands require a broader attention focus than tasks low in task complexity and work pressure. As such, we hypothesize that the state neuroticism- momentary task performance relationship will be less curvilinear for tasks high in momentary job demands than for tasks low in momentary job demands. The reasoning behind this is that, because of their high demanding nature with many relevant task cues, tasks high in momentary job demands require a broader attention focus, which is in line with the findings of Easterbrook [24], who found that a lower emotional level (i.e., lower state neuroticism) is required for tasks involving a wide range of peripheral cues. On the contrary, higher levels of state neuroticism matter more for tasks low in job demands because low complex tasks or tasks that have to be carried out under low work pressure demand a higher level of attention resources. As such, the optimal task performance level for complex, high-pressure tasks is located at lower levels of state neuroticism, while it is located at moderate levels of state neuroticism for low complex, low-pressure tasks. This reflects itself in a stronger curvilinear relationship (i.e., inverted U-shaped) for tasks low in job demands. This leads to the following hypothesis:

#### Hypothesis 2

The state neuroticism-performance link will be moderated by work pressure and task complexity in the sense that for low complex, low pressure tasks the state neuroticism-performance link will be more curvilinear than for complex, high pressure tasks.

To study the curvilinear within-person relationship between state neuroticism and task performance, as well as the moderating effect of within-person fluctuations in job demands, one diary study and two experiences sampling studies were conducted.

## Method

### Ethics statement

For the three studies no formal ethical committee statements were obtained because the research techniques were non-invasive and harmless. We did discuss the possible ethical issues related to this research elaborately within the research units of the respective authors. Organizations were contacted to participate in one of the three studies. Organizations that agreed to participate first asked the individual employees whether they were willing to participate in the study and if this was the case, they provided us with their individual contact details. The authors had access to these contact details (i.e., email addresses) for two reasons only, namely to send the questionnaires to the participants and to link the repeated measurements to the same individual. The data were treated confidentially in every step of the research process. Prior to carrying out the analyses, data were fully anonymized. Participants were also given an oral informed consent (not a written one) including the general aim of the studies, which clearly mentioned the possibility to withdraw from participating in the study.

### Sample and procedure

#### Study 1

Respondents were 45 employees from the administrative headquarters of a large retail company, of which 19 were men. The average age of the respondents was 33.8 years (*SD* = 5.9) and their average organization tenure was 7.7 years (*SD* = 6.5). The sample consisted of both entry-level workers and more seasoned employees with leadership responsibilities. All of the employees in the first study held white-collar jobs.

Data were collected via an online survey system. First, participants completed the NEO-FFI [35]. One week later they took part in a 10-day lasting daily diary study during which they had to fill out a questionnaire just before they left the office. This resulted in 277 out of a maximum of 450 (45 employees ×10 days) data points, or a response rate of 61.6%. In the diary study participants had to recall and report on a task they carried out on a specific hour during the day. These specific time slots of one hour ranged from nine in the morning until four in the afternoon. Participants had to report on a different, random time slot every day. To help participants reconstruct the task they were executing during that specific time slot, they were asked to describe the task (see [36]). In addition, they also rated the level of momentary task complexity, neuroticism, and task performance.

#### Study 2

In the second study, 52 employees took part. Thirty of them were men. The average age of the participants was 32.2 years (*SD* = 9.3) and their average company tenure totaled 6.1 years (*SD* = 8.7). The majority of respondents were employed in the telecom and ICT sector (46.2%), and in media, entertainment, and communication (17.3%). Most of them were in an early career stage as the median age of our sample was 29 years and the median job tenure was 3 years.

An online survey system was used to collect data. First, participants completed the NEO-FFI [36]. One week later, and for five consecutive workdays, employees received two electronic questionnaires before noon and two in the afternoon. The first questionnaire was sent at a random moment during the workday (once before noon and once in the afternoon) and asked about the momentary level of neuroticism and work pressure. The second questionnaire, sent one hour after the first, asked the participants to rate their momentary task performance. Note that, because of the built-in time lag between state neuroticism and momentary task performance, our results allow to test directionality of the state neuroticism - momentary task performance relationship. This data collection procedure resulted in 324 dyadic responses (including personality, work pressure, and performance) out of a maximum of 520 possible responses (52 participants ×10 measuring moments), or a response rate of 62.3%.

#### Study 3

Respondents were 130 employees working for a large company in the financial sector. Respondents were mainly administrative staff and their managers. Of these 130 employees, 60% were female. The average age of the respondents was 39.3 years (*SD* = 10.8) and their average organization tenure was 14.4 years (*SD* = 12.7).

The data collection was similar to Study 2, except for the fact that we used the Mini-Markers scale [37] instead of the NEO-FFI [35], and that the study spanned 10 instead of five consecutive working days. In addition to work pressure, we also measured task complexity. This resulted in 1170 dyadic responses out of a maximum of 2600 responses (130 participants ×20 measuring moments), or a response rate of 45.0%.

### Measures

*Task performance* was measured in the three studies by the seven-item task performance subscale of Williams and Anderson [38]. Items were adapted to allow for a self-rated momentary assessment of task performance. One of the items was “Since completing the previous survey I adequately completed assigned duties”. The seven items were answered on a seven-point rating scale in Study 1 and Study 3, while a five-point rating scale was used in Study 2. In all cases, the rating scale ranged from “completely disagree” to “completely agree”. Alpha reliability coefficients were calculated for each measurement moment separately (see [39]). For task performance, alpha reliability coefficients ranged between.34 and.82 for Study 1, between.79 and.90 for Study 2, and between.77 and.89 for Study 3. The mean alpha reliability coefficients were.71 (*SD* = .14) for Study 1,.86 (*SD* = .04) for Study 2, and.83 (*SD* = .04) for Study 3.

*State neuroticism* was measured with the 12 corresponding items of the NEO-FFI [36] in studies 1 and 2. The items were adapted to allow for momentary measurement (e.g. “When carrying out this task, I got mad about the way in which people treated me”). The items were answered using a five-point rating scale, ranging from “completely disagree” to “completely agree”. The alpha reliability coefficients for state neuroticism ranged between.66 and.88 for Study 1 and between.81 and.92 for Study 2. The average alpha reliability coefficient for state neuroticism was.77 (*SD* = .08) in Study 1 and.88 (*SD* = .03) in Study 2. In Study 3, state neuroticism was measured using the eight adjectives of the Mini-Markers scale [37]. The instructions were adapted to enable momentary measurement. All items were answered using a seven-point scale, ranging from “not at all applicable” to “extremely applicable”. The alpha reliability coefficient ranged between.65 and.85, while the mean alpha coefficient was.77 (*SD* = .06).

*Task complexity* was measured in Study 1 using a single-item scale (i.e., “Indicate how complex this task was”) accompanied by a seven-point rating scale ranging from “very low in complexity” to “very high in complexity”. In Study 3, task complexity was measured using the four items of the subjective task complexity scale of Maynard and Hakel [40]. Instructions were adapted to allow for momentary measurement (e.g. “The task I am currently working on is complex”). The four items were answered using a seven-point scale, ranging from “completely disagree” to “completely agree”. The alpha reliability coefficient for subjective task complexity ranged between.88 and.96, and the mean alpha reliability coefficient was.93 (*SD* = .02).

*Work pressure* was measured in studies 2 and 3 using three items of the questionnaire by van Veldhoven and colleagues [41]. An example item is “I had to work really fast on the task I was doing”. The items had to be answered on a five-point rating scale, ranging from completely disagree to completely agree. The alpha reliability coefficients ranged between.55 and.86 for Study 2 and between.79 and.93 for Study 3. The mean alpha coefficient was.73 (*SD* = .10) for Study 2 and.89 (*SD* = .04) for Study 3.

### Analyses

Because participants in the diary study provided ratings on 10 consecutive working days, the data have a nested structure with *i* measurements nested within *j* persons. To account for this nested data structure, we analyzed the data using a two-level regression model with measurements at the first and persons at the second level. For the two experience sampling studies (i.e., studies 2 and 3), *i* measurements were nested within *j* days, which in turn were nested within *k* persons. Therefore, we analyzed the data using three-level regression analyses. Analyses were done using the lme4 package in R [42]. Because all our hypotheses pertain to the within-person level, we group-mean centered (or person-centered) all predictor variables (i.e., state neuroticism, work pressure, and task complexity) before conducting the analyses. By doing so, we removed all between-person variability from the predictors.

In all analyses, we first tested an intercept-only model in which momentary task performance was predicted by a random intercept for the persons (for the diary study) or random intercepts for persons and days (for the two experience sampling studies). These random intercept models allowed us to estimate the amount of variance in task performance ratings at the different levels of the model (i.e., person, day, and momentary level).

Second, we added the group-mean centered state neuroticism scores and the squared effect of the group-mean centered state neuroticism scores to the model. To test whether the effects of state neuroticism and state neuroticism squared on momentary task performance varied across persons (for all three studies) and days (for the two experience sampling studies), we tested whether the slopes of state neuroticism and state neuroticism squared were fixed or random. This was done by testing each slope individually for randomness. For example, to test whether the effect of state neuroticism varied across persons, we tested whether a model with a random slope for state neuroticism on the person-level fitted our data significantly better than a model without random slopes. Similarly, to test whether the effect of state neuroticism squared varied across days, we tested whether a model with a random slope for state neuroticism squared on the day-level gave a significantly better fit than a model without random slopes. To test whether the model with the random slope fitted significantly better than the model without random slopes, we compared them using a log-likelihood difference test. For reasons of parsimony, statistically significant random effects (*p*<.05) were included in the final model whereas non-significant random slopes were trimmed [43].

Finally, we added the main effect of the group-mean centered task complexity/work pressure scores and the interactions between the group-mean centered task complexity/work pressure scores and group-mean centered state neuroticism and state neuroticism squared to the model. Again, random effects were tested on a one-by-one basis, both on the person (for all studies) and the day-level (for the two experience sampling studies). For task complexity/work pressure this implied comparing a model with a random slope for task complexity/work pressure to a model without random slopes. For the interactions, the test was different because it is meaningless to model an interaction without modeling the main effects. Therefore, we compared a model with random slopes for the two main effects to a model with random slopes for the main effects plus the interaction. Again, random slopes were tested by comparing the models using a log-likelihood difference test with *p*<.05. Non-significant random slopes were again excluded from the final model [43].

## Results

Descriptive statistics (i.e., means, standard deviations, intra-class correlations, and correlations between the person-centered variables) for Study 1, Study 2, and Study 3 are shown in Table 1, Table 2, and Table 3, respectively. When reviewing the results of the multilevel regression models we only discuss the fixed effects. The reason is that our hypotheses pertain to the fixed part of the models only. Random effects were only included because misspecification of the random part can affect the parameter estimates and standard errors of the fixed part [44].

**Table 1:** Means, standard deviations, intra-class correlations and correlations for trait neuroticism, state neuroticism, task performance, and task complexity (Study 1).

	*M*	*SD*	ICC_person_	ICC_day_	1.	2.	3.
1. Trait neuroticism	2.76	.63	1.00	-			
2. State neuroticism	2.03	.76	.50	.50	.41**		
3. Task performance	5.67	.73	.23	.77	−.14	−.66**	
4. Task complexity	3.77	1.67	.25	.75	−.30†	.11	−.10

**p<.01;

*p<.05;

† p<.10. All variables were person-centered before computing the correlations.

**Table 2:** Means, standard deviations, intra-class correlations and correlations for trait neuroticism, state neuroticism, task performance, and work pressure (Study 2).

	*M*	*SD*	ICC_person_	ICC_day_	ICC_moment_	1.	2.	3.
1. Trait neuroticism	2.45	.70	1.00	-	-			
2. State neuroticism	1.76	.66	.39	.13	.48	.43**		
3. Task performance	4.13	.67	.37	.22	.41	.03	−.14	
4. Work pressure	2.93	.90	.47	.09	.44	.35*	.36*	.14

**p<.01;

*p<.05;

† . All variables were person-centered before computing the correlations.

**Table 3:** Means, standard deviations, intra-class correlations and correlations for trait neuroticism, state neuroticism, task performance, work pressure and task complexity (Study 3).

	*M*	*SD*	ICC_person_	ICC_day_	ICC_moment_	1.	2.	3.	4.
1. Trait neuroticism	2.99	.77	1.00	-	-				
2. State neuroticism	2.55	.89	.63	.11	.26	.64**			
3. Task performance	5.68	.88	.50	.12	.38	−.39**	−.49**		
4. Work pressure	4.30	1.59	.62	.14	.24	.11	.22*	−.06	
5. Task complexity	4.22	1.54	.57	.12	.31	−.07	−.04	.12	.52**

**p<.01;

*p<.05. All variables were person-centered before computing the correlations.

### Study 1

First, we tested an intercept-only model (see Table 4). This allowed us to estimate the amount of variance in momentary task performance ratings that is attributable to the person and the moment level respectively. Results showed that 23% of the variance in momentary task performance was due to between-person differences, while 77% of the variance was situated at the momentary level. In other words, the intra-class correlation coefficients revealed that only a small amount of the total variance in momentary task performance could be explained by between-person differences, while the large majority of the variation was within-person variation.

**Table 4:** Parameter estimates and variance components of the HLM models tested (Study 1).

	Fixed effects											Random effects		
Model equations	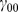	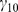	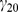	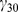	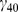	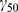	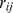	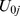	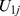	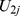	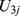		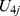	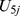
*Intercept-only model (empty model)*														
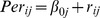	5.67**	-	-	-	-	-	.42	.13	-	-	-		-	-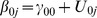														
*Model 1a*														
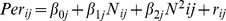	5.67**	−.44**	-.03	-	-	-	.33	.15	.17	*ns*	-		-	-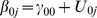														
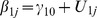														
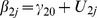														
*Model 1b*														
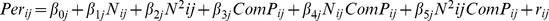	5.66**	−.48**	.04	.05	−.04	.11^†^	.30	.16	.14	*ns*	.02		*ns*	*Ns*
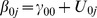														
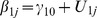														
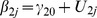														
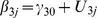														
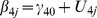														
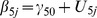														

*Note:* **p<.01 (two-tailed); *p<.05 (two-tailed); ^†^ *p*<.10 (two-tailed).

Per  =  performance; Comp  =  task complexity; N =  neuroticism.

In a second step, state neuroticism and the squared effect of state neuroticism were added to the model (see Table 4 – Model 1a). The results of this analysis showed that state neuroticism was negatively related to momentary task performance (*γ*_10_ = −.44, *p*<.001), while no evidence was found for a curvilinear relationship (*γ*_20_ = −.03, *p* = .767). As a result, Hypothesis 1 was not supported.

Third, task complexity and the interactions between task complexity and the linear and quadratic effect of state neuroticism were added to the model to test if this job demand moderated the linear and quadratic relationship between state neuroticism and momentary task performance. The results of this analysis (see Model 1b in Table 4) showed no significant moderation effect of task complexity on the linear state neuroticism – momentary task performance relationship (*γ*_40_ = −.04, *p* = .531), nor on the quadratic one (*γ*_50_ = .11, *p* = .085).

**Figure 1:**
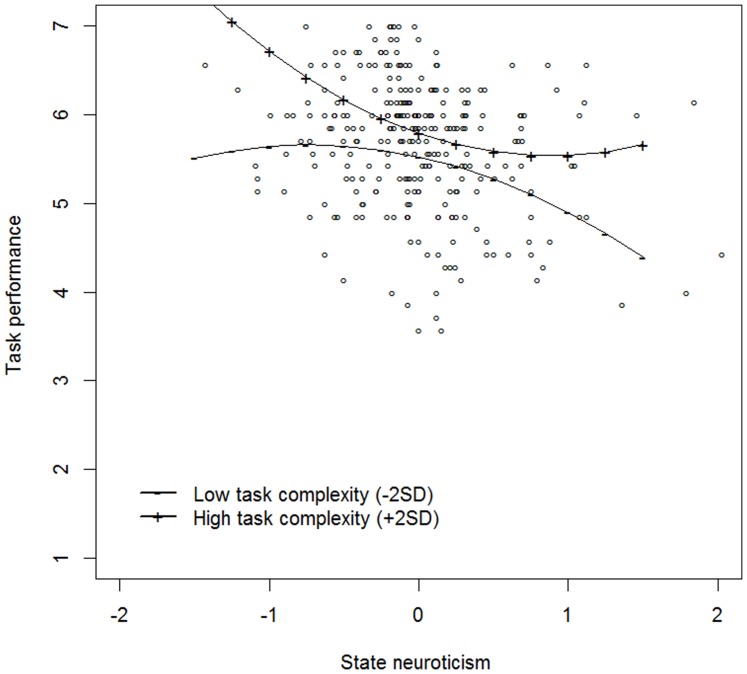
The moderating effect of low (−2 *SD*) and high (+2 *SD*) levels of momentary task complexity on the state neuroticism – task performance relationship (Study 1).

Finally, we plotted the relationship between state neuroticism and momentary task performance as a function of low (mean – 2 *SD*) and high (mean +2 *SD*) levels of momentary task complexity. As can be seen in Figure 1, for both high and low task complexity the state neuroticism-momentary task performance relationship showed a trend towards curvilinearity. In particular, whereas the state neuroticism-momentary task performance relationship showed an exponential decreasing curve for tasks high in complexity, an inverted U-shaped curve emerged for tasks low in complexity. However, because these effects were not statistically significant at *p*<.05 (i.e., the *p*-value of the quadratic moderation effect was.085), they have to be interpreted with caution and their replication in independent samples is of key importance.

### Study 2

The intercept-only model (see Table 5) revealed that 37% of the variance in momentary task performance in Study 2 was due to between-person differences, 22% to between-day differences, and 41% of the variance was situated at the momentary level. This again demonstrated that there is a substantial amount of within-person variability in momentary task performance.

**Table 5:** Parameter estimates and variance components of the HLM models tested (Study 2).

	Fixed effects															Random effects				
Model equations	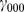	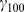	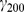	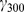	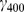	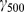	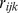	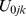	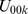	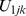	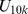	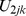	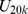	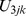	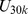		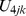	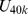	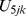	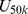
*Intercept-only model (empty model)*																				
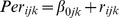	4.12**	-	-	-	-	-	.19	.10	.17	-	-	-	-	-	-		-	-	-	-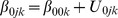																				
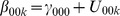																				
*Model 1a*																				
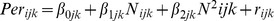	4.11**	−.20**	.03	-	-	-	.17	.12	.16	*ns*	*ns*	*ns*	*ns*	-	-		-	-	-	-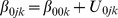																				
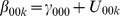																				
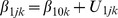																				
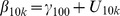																				
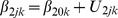																				
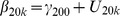																				
*Model 1b*																				
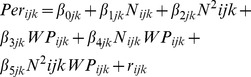	4.10**	−.27**	.16	−.10	−.20	.34*	.15	.04	.19	*ns*	*ns*	*ns*	*ns*	.27	*ns*		-	*-*	-	*-*
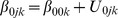																				
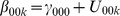																				
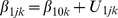																				
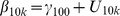																				
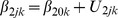																				
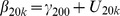																				
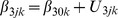																				
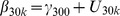																				
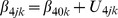																				
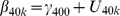																				
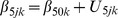																				
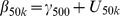																				

*Note:* **p<.01 (two-tailed); *p<.05 (two-tailed); ^†^ *p*<.10 (two-tailed); Per  =  performance; WP =  work pressure; N =  neuroticism.

In a second step, state neuroticism and its squared effect were added to the model (see model 1a – Table 5). Results were similar to those of the first study in that we found evidence for a significant, negative linear effect between state neuroticism and momentary task performance (γ_100_ = −.20, *p* = .008), while no quadratic effect was found (γ_200_ = .03, *p* = .749). Hence, Hypothesis 1 could not be supported.

Third, the moderating effect of work pressure on the linear and quadratic state neuroticism-momentary task performance relationship was tested by adding work pressure and the interactions between work pressure and the linear and squared effect of state neuroticism to the model (see model 1b in Table 5). A significant moderation effect of work pressure on the quadratic state neuroticism-momentary task performance relationship (γ_500_ = .34, *p* = .033) was found, whereas there was no moderation effect on the linear relationship (γ_400_ = −.20, *p* = .184). Similar to the tentative trend that was found in Study 1, the curvilinearity of the state neuroticism-momentary task performance relationship depended on the momentary job demands, thereby lending support to Hypothesis 2.

**Figure 2:**
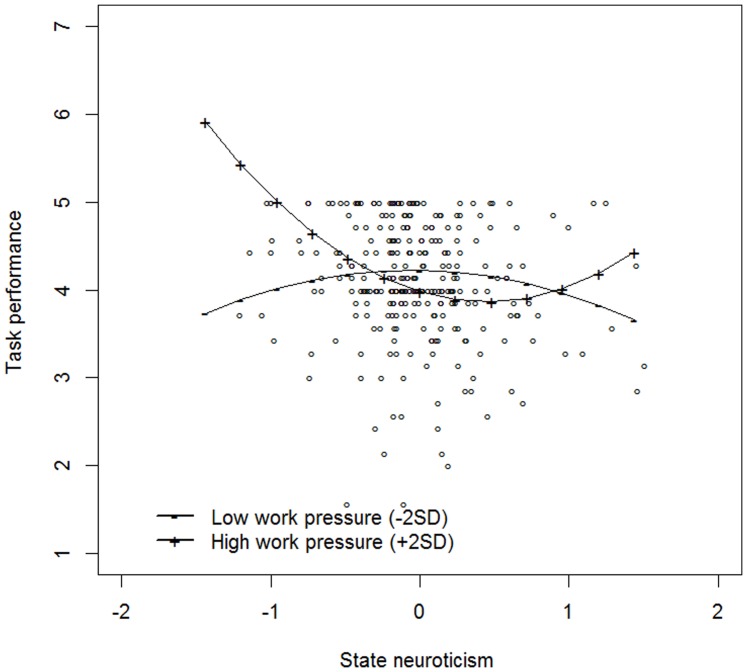
The moderating effect of low (−2 *SD*) and high (+2 *SD*) levels of momentary work pressure on the state neuroticism – task performance relationship (Study 2).

To inspect the nature of this moderation effect, we again plotted the relationship between neuroticism and task performance as a function of low (mean – 2 *SD*) and high (mean +2 *SD*) levels of work pressure. From Figure 2, it can be seen that the relationship for both high and low work pressure was very similar to the one we found in Study 1. In particular, for high levels of work pressure, the state neuroticism-momentary task performance relationship was best described by an exponential decreasing curve, while for low levels, an inverted U-shaped relationship was observed.

### Study 3

Similar to the first and second study, in a first step, intra-class correlation coefficients were calculated to assess the amount of between- and within-person variability in momentary task performance. In this third study 50% of the variance in momentary task performance was attributable to the between-person level, 12% to the day-level, and 38% of the variance was situated at the momentary level. Again, a large part of the variation was situated at the within-person level.

In accordance with the first and second study, we found a significant linear effect of state neuroticism on momentary task performance (γ_100_ = −.11, *p* = .029), while the quadratic effect was non-significant (γ_200_ = .05, *p* = .207) (see Model 1a – Table 6). These findings once again did not support the first hypothesis.

**Table 6:** Parameter estimates and variance components of the HLM models tested (Study 3)

	Fixed effects															Random effects				
Model equations	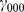	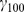	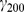	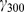	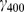	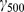	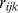	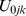	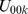	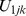	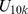	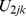	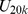	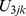	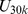		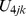	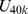	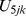	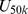
*Intercept-only model (empty model)*																				
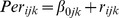	5.61**	-	-	-	-	-	.29	.09	.38	-	-	-	-	-	-		-	-	-	-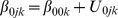																				
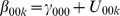																				
*Model 1a*																				
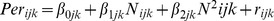	5.62**	−.11*	.05	-	-	-	.24	.08	.42	.28	.00	.06	*ns*	-	-		-	-	-	-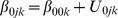																				
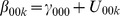																				
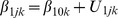																				
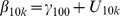																				
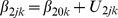																				
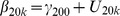																				
*Model 1b*																				
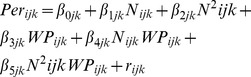	5.62**	−.12**	.06	−.05^†^	−.08^†^	.05	.21	.03	.43	.21	.00	.04	*ns*	.08	.00		*ns*	.00	*ns*	*ns*
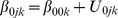																				
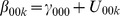																				
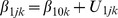																				
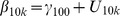																				
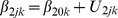																				
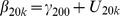																				
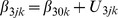																				
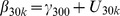																				
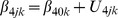																				
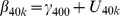																				
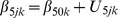																				
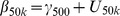																				
*Model 1c*																				
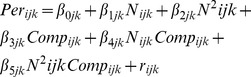	5.62**	−.11*	.03	−.03	−.10*	.03	.21	.06	.44	.21	.00	.05	*ns*	.05	.01		*ns*	.01	*ns*	*ns*
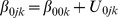																				
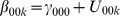																				
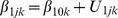																				
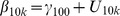																				
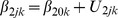																				
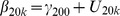																				
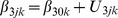																				
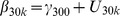																				
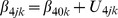																				
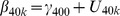																				
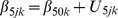																				
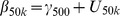																				

*Note:* **p<.01 (two-tailed); *p<.05 (two-tailed); ^†^ *p*<.10 (two-tailed).

Per  =  performance; WP =  work pressure; Comp  =  task complexity; N =  neuroticism.

Third, when testing the moderating effect of work pressure (Model 1b – Table 6), no moderating effect on the linear (γ_400_ = −.08, *p* = .082), nor on the quadratic (γ_500_ = .05, *p* = .114) state neuroticism-momentary task performance relationship was found. Nevertheless, the results revealed a trend towards a moderation of the linear state neuroticism-momentary task performance relationship. For task complexity, we found a significant moderation effect on the linear (γ_400_ = −.10, *p* = .025), but not on the quadratic effect of state neuroticism (γ_500_ = .04, *p* = .186). Thus, contrary to the two aforementioned studies, no support was found for Hypothesis 2.

**Figure 3:**
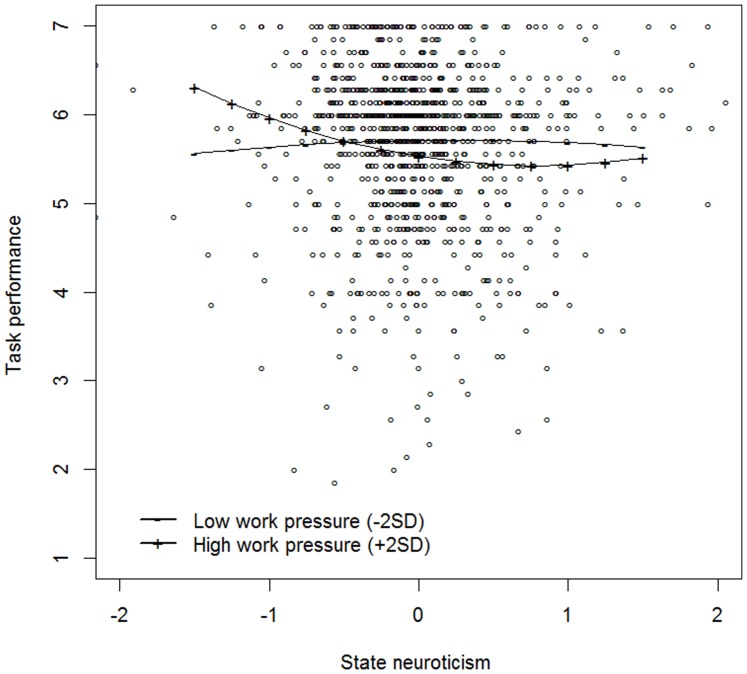
The moderating effect of low (−2 *SD*) and high (+2 *SD*) levels of momentary work pressure on the state neuroticism – task performance relationship (Study 3).

**Figure 4:**
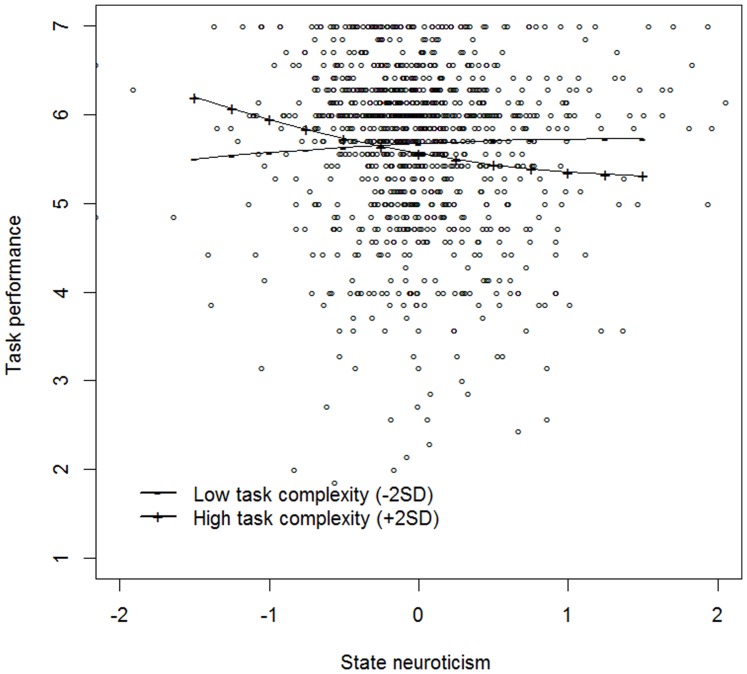
The moderating effect of low (−2 *SD*) and high (+2 *SD*) levels of momentary task complexity on the state neuroticism – task performance relationship (Study 3).

To inspect the nature of the moderation effects of work pressure and task complexity, we plotted the relationship between state neuroticism and momentary task performance as a function of low (mean – 2 *SD*) and high (mean + 2 *SD*) levels of work pressure and task complexity. From Figure 3 and Figure 4, it can be seen that there is only a negative state neuroticism-momentary task performance relationship for tasks high in work pressure and high in task complexity.

## Discussion

Whereas our findings revealed that the within-person state neuroticism-momentary task performance relationship is generally linear, we found tentative support for a curvilinear relationship when we took into account the moderating effects of within-person fluctuations in momentary job demands. Combined with the finding that a large part of the variation in task performance is situated at the within-person level, this finding is an important one as it invigorates the crucial importance of a recent call in the literature to study the effects of moderators on the within-person personality-performance relationship [33]. It is also important to note that only in one of the three studies strong support for curvilinearity was found, while in our first study a trend towards curvilinearity was observed. Whereas such mixed findings were not expected, they parallel previous findings at the trait level, where some studies did [2], and others did not find a curvilinear relationship [10], [45]. In what follows, we will elaborate on the theoretical and practical implications of these findings.

### Theoretical implications

When tasks were carried out under high momentary job demands (i.e., task complexity or work pressure), the relationship between state neuroticism and momentary task performance showed an exponential decreasing curve in one of the three studies (and a similar trend in another study). Yet, this relationship reversed (i.e., an inverted U-shaped curve) when momentary job demands were low. In other words, low levels of state neuroticism were associated with high levels of momentary task performance under high job demands (that is, when the job demands are higher than normally), whereas under low job demands (i.e., job demands lower that normally) an average level of state neuroticism appeared to be most beneficial.

The reason is that tasks high in momentary job demands are typically characterized by a large number of relevant task cues. Therefore, the attention focus cannot be too narrow, as one would neglect at least some of the relevant task cues, which would in turn deteriorate task performance. Instead, low levels of state neuroticism, which are associated with a broad attention focus, are in these circumstances most beneficial. Conversely, in the case of low job demands, there are typically only few cues that need to be taken into account. Therefore, moderate levels of state neuroticism are optimal as they help people to focus their attention on the few relevant task cues. Indeed, low levels of state neuroticism would result in an attention focus that is too broad, while the attention focus would be too narrow for high levels of state neuroticism, with both cases being detrimental for task performance (see [2]).

The key role of the moderators (i.e., the momentary job demands) is obvious from the fact that when they are not taken into account, the relationship between state neuroticism and momentary task performance is linear in all studies. Yet, whereas such a linear relationship may be optimal in a statistical sense, our results also show that in some cases these linear relationships fail to capture the processes underlying the relationship between state neuroticism and momentary task performance because they are unable to describe what happens when job demands deviate from their habitual level. That is, the linear relationship only results from the aggregation of two opposite curvilinear relationships. As such, our findings support the recent call for more research on the effects of moderators of the personality-performance relationship [33].

Whereas a curvilinear state neuroticism-momentary task performance relationship was found in the second study and a trend towards a quadratic effect was observed in the first study, this was not the case in the third one. A first possible reason is the use of a different state neuroticism measure. In the first two studies, the neuroticism subscale of the NEO-FFI [35] was used, while in the third study state neuroticism was measured using the Mini-Markers [37]. As Zillig and colleagues [46] have demonstrated, the major difference between both scales is that the Mini-Markers tap more into the behavioral and cognitive components, while the NEO-FFI primarily measures the affective component of neuroticism. As it is particularly the affective component that relates to attention focus [24], the curvilinear state neuroticism-task performance relationship may only show up for scales that thoroughly capture this component. A second possible reason for the differences between studies is that the samples differed substantially with regard to job content. More specifically, the third sample was different from the first two in that participants were financial professionals whose jobs are known for their high levels of work pressure and task complexity. Despite the fact that also in this sample of employees there was substantial within-person variability in the levels of work pressure and task complexity, the financial professionals often found themselves at the high ends of the work pressure and task complexity continuum. As a result, for this specific group of people, within-person fluctuations in state neuroticism might not have had the same effect on momentary task performance than for people who generally experience less extreme levels of work pressure and task complexity (i.e., the employees in studies 1 and 2). Note by the way that this account might explain the linear relationship that was found in Study 3 as according to Hypothesis 2 the relationship between state neuroticism and momentary task performance should be less curvilinear (and thus more linear) under high levels of work pressure and task complexity. Finally, despite the differences between the three studies, also in the third study task complexity moderated the relationship between neuroticism and momentary task performance, thereby again providing support for the crucial role of within-person fluctuations in the work environment as a moderator of the state neuroticism-momentary task performance relationship.

When comparing our findings with those of Le and colleagues [2], we see that different patterns of findings emerge at the within- and at the between-person level. First, Le and colleagues [2] found consistent support for a curvilinear relationship between neuroticism and task performance at the between-person level, whereas we only found a curvilinear relationship between state neuroticism and momentary levels of task performance when taking into account the moderating role of momentary job demands. Second, while Le et al. [2] repeatedly found a moderating effect of between-person differences in job demands (in their case work pressure) on the curvilinear relationship between trait neuroticism and general task performance, we only found a statistically significant moderating effect in one out of three studies, together with a clear trend in another one (i.e., Study 1). Third, Le and colleagues [2] found that an inverted U-shape best described their data (both for high and low levels of job demands). In contrast, we found an inverted U-shaped relationship between state neuroticism and momentary task performance only for low momentary levels of job demands (i.e., work pressure), while for high levels of momentary work pressure an exponential decreasing curve best described the relationship between state neuroticism and momentary task performance. Together, these results tend to suggest that the findings at the between-person level cannot be readily transferred to the within-person level.

### Practical implications

Given the importance of both the personality and situational component, managers and HR-experts should not only focus on the personality component, but should pay an equal amount of attention to the job circumstances when assessing employees. This is important, as an employee who has to work under high job demands will most likely perform best when he/she is low in state neuroticism. However, when this same employee is selected into a job with low job demands he/she will probably perform less than someone who shows moderate levels of state neuroticism. Therefore, when evaluating candidates one needs to assess which levels of job demands the function holds, as well as the amount of state neuroticism the employee shows under these conditions.

The insights provided in this paper can also find their application outside assessment and selection. Looking at these findings from a job redesign perspective one could conclude that job circumstances can be altered to fit the personality of the employee better. For example, the task performance of an employee experiencing moderate levels of state neuroticism under high job demands can be improved by lowering the job demands he/she faces on a day-to-day level. Instead, for an employee who experiences low levels of state neuroticism, a challenging environment with high levels of job demands might be most beneficial.

### Limitations and further research

All study variables were measured using self-reports. Whereas in typical, cross-sectional designs this may cause validity problems because of self-serving biases, self-reports are less problematic when the focus is on within-person differences [32], [47]. The reason is that in that case the individual's responses are evaluated relative to the individual's average response, which implies that individual differences in self-serving bias are removed from the data [48]. For example, when a person systematically overestimates his/her level of state neuroticism with one unit, the average state neuroticism score will also be inflated with one unit. As a result, the person-centered scores (i.e., state neuroticism of person *i* – average state neuroticism of person *i*) will no longer contain the one-unit inflation and as a result they will no longer contain the self-serving bias. Because also task complexity and work pressure scores were group-mean centered (or person-centered) in all analyses, all between-person and between-organization differences in task complexity and work pressure were removed from the data. Note that the strategy of group-mean centering (or person-centering) was only applied to the predictor variables (i.e., state neuroticism, momentary work pressure and momentary task complexity). For the criterion (i.e., momentary task performance), self-serving biases were removed from the data by estimating the person-specific (or random) intercepts in the multilevel regression models. By doing so, the multilevel regression models modeled deviations of the momentary task performance ratings from the person-specific average (i.e., the average momentary task performance for that individual), rather than the raw momentary task performance scores. Also note that the reasoning concerning the separation of within- and between-variability does not hold in typical, cross-sectional designs because in these designs individual differences in self-serving bias are confounded with individual differences in the variable under study.

Second, no causal inferences could be made about the state neuroticism-momentary task performance relationship. Study 2 and Study 3, however, did allow us to determine the directionality of this relationship because of the built-in time lags in the experience sampling studies. In particular, because state neuroticism was measured one hour before the measurement of momentary task performance, we can conclude that state neuroticism at time *t* was related to momentary task performance at time *t+1* in both experience sampling studies. For reasons of completeness, we also tested the reversed effect, that is, the relationship between momentary task performance at time *t-1* and state neuroticism at time *t* in Study 2 and Study 3. In Study 2, we found a non-significant time-lagged effect of momentary task performance on state neuroticism (*γ* = .01, *p* = .95), while in the third study this effect was positive and statistically significant (*γ* = .24, *p*<.01). Whereas the findings are not consistent, the positive relationship between momentary task performance at time *t-1* and state neuroticism at time *t* in Study 3 hints at the existence of a bidirectional relationship between state neuroticism and momentary task performance. Note that such a bidirectional relationship is not that surprising given the theoretical model of Judge and colleagues (i.e., CSEJAM model) [49], which explicitly states that the performance of an employee can influence his/her personality-related states. However, given the inconsistency of our findings at this point, we consider this issue an interesting avenue for further research.

In contrast to Le et al. [2] we did not find a direct curvilinear relationship between state neuroticism and momentary task performance. One possible reason for not finding this hypothesized curvilinear relationship may be low statistical power for the quadratic effects. If statistical power is too low, these curvilinear effects will not be detected even when they exist. To reassure us from the fact that statistical power issues were not responsible for our findings, we carried out a post-hoc power simulation study for each of the three studies using the MLPowSim software [50]. As input for these simulation studies we used the parameter estimates found in each of our three different studies. Moreover, we used the number of participants as our level 3 input (i.e., 45 for Study 1, 52 for Study 2 and 130 for Study 3), the mean number of days that participants filled in the questionnaire as level 2 input (i.e., 6 days for Study 1, 4 days for Study 2 and 5 days for Study 3) and the mean number of moments that where filled in by participants as level 1 input (i.e., 6 moments for Study 2 and 9 moments for Study 3). The results of these power calculations (also see Table 7) showed that in Study 1 there was low statistical power for the quadratic effect of state neuroticism (1-*β* = .14), the effect of task complexity (1-*β* = .50), and the interaction effect of state neuroticism and task complexity (1-*β* = .23) on momentary task performance. For Study 2 and Study 3, however, there was sufficient statistical power to detect the hypothesized effects for all studied variables (1-*β*>.95 for all variables). In other words, statistical power issues can only account for the absence of the quadratic effect in Study 1.

**Table 7:** Post-hoc power calculations for all three studies, using the MLPowSim-software.

	Study 1	Study 2	Study 3
State N	1.00	1.00	1.00
State N^2^	.14	1.00	1.00
Task complexity	.50	-	1.00
Work pressure	-	1.00	1.00
State N× Task complexity	.23	-	1.00
State N^2^× Task complexity	.91	-	1.00
State N× Work pressure	-	1.00	1.00
State N^2^× Work pressure	-	1.00	1.00

*Note*: N =  neuroticism; N^2^ =  quadratic effect of state neuroticism.

While our results emphasized the importance of taking into account momentary job characteristics (i.e., task complexity and work pressure) when studying the state neuroticism-momentary task performance relationship, they were limited to two job demands and one personality dimension only. Further research should incorporate other possible moderators as well as other personality dimensions as Yang and colleagues [51] showed that different personality dimensions will likely be influenced by different situational characteristics.

Another, related, avenue for future research is to investigate the linear and curvilinear effects of the different neuroticism facets. It is, for example, possible that the shape of the relationship between (state) depression and momentary task performance will differ from that of (state) anxiety and momentary task performance. Our data, however, do not allow testing these relationships as we used the NEO-FFI to measure state neuroticism, and this shortened version of the NEO-PI-R does not cover all six neuroticism facets. Based on the work of Saucier [52] we were however able to extract three facets from the NEO-FFI, namely anxiety, depression and self-reproach. According to Saucier [52], anxiety and depression are closely related to negative affect, while self-reproach encompasses more self-conscious negative emotions, such as embarrassment, shame and guilt. We tested curvilinearity for the three facets of neuroticism separately and found a moderating effect of momentary task complexity on the curvilinear relationship between state depression and momentary task performance in Study 1 and a moderating effect of momentary work pressure on the curvilinear relationships between state anxiety and momentary task performance, and between state self-reproach and momentary task performance in Study 2. These preliminary findings indicate that not all neuroticism facets need to be related to momentary task performance in the same way. However, given the inconsistent nature of these preliminary findings, future research needs to be conducted with personality measures (e.g., the NEO-PI-R) that capture all six facets of the state neuroticism dimension.

## Conclusions

With this study we provided new insights into the neuroticism-task performance relationship. First, we showed that a substantial part of the variation in neuroticism and task performance is located within the individual, a finding that underscores the importance of studying these within-person components. Second, we found that the within-person state neuroticism-momentary task performance relationship is generally linear, although tentative support was found for a curvilinear relationship when taking into account the moderating effects of momentary job demands in two of the three studies. Finally, results from all three studies emphasized the importance of taking into account the moderating effects momentary job demands have on the state neuroticism-momentary task performance relationship. In particular, low levels of state neuroticism appeared to be most beneficial in high demanding tasks, whereas more moderate levels of state neuroticism were optimal under low momentary job demands.
