# Correction: The Curvilinear Relationship Between State Neuroticism and Momentary Task Performance

**DOI:** 10.1371/journal.pone.0114538

**Published:** 2014-11-21

**Authors:** The PLOS ONE Staff

Multiple rows have been removed from Table 4 due to a typesetting error. The publisher apologizes for the error. Please view the correct Table 4 here.

**Table 4:** Parameter estimates and variance components of the HLM models tested (Study 1).

	Fixed effects						Random effects						
Model equations	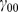	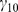	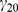	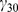	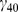	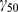	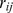	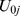	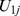	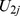	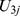	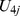	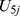
*Intercept-only model (empty model)*													
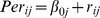	5.67^**^	-	-	-	-	-	.42	.13	-	-	-	-	-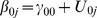													
*Model 1a*													
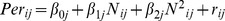	5.67^**^	−.44^**^	−.03	-	-	-	.33	.15	.17	*ns*	-	-	-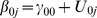													
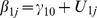													
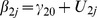													
*Model 1b*													
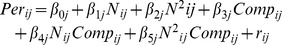	5.66^**^	−.48^**^	.04	.05	−.04	.11†	.30	.16	.14	*ns*	.02	*ns*	*ns*
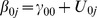													
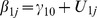													
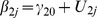													
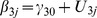													
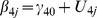													
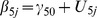													

*Note:* ^**^ *p*<.01 (two-tailed);

^*^ *p*<.05 (two-tailed);

† *p*<.10 (two-tailed).

Per  =  performance; Comp  =  task complexity; N  =  neuroticism.
